# Completion of Treatment Escalation Plans and Resuscitation Discussions in Frail and Elderly Patients With Hip Fractures

**DOI:** 10.7759/cureus.106298

**Published:** 2026-04-01

**Authors:** Henry Stonelake, Neil Ashwood

**Affiliations:** 1 Trauma and Orthopaedics, University Hospitals of Derby and Burton NHS Trust, Derby, GBR

**Keywords:** fragility hip fracture, orthogeriatric medicine, orthopaedics trauma, recommended summary plan for emergency care and treatment (respect), service evaluation and improvement, treatment escalation plan

## Abstract

Background

Hip fractures disproportionately occur in frail and elderly patients and carry high mortality even with optimal management. The British Orthopaedic Association recommends that all frail and elderly trauma patients have ceilings of treatment (CoTs) and appropriateness of cardiopulmonary resuscitation (CPR) decided pre-operatively. Our hospital documents these treatment escalation plans (TEPs) within the Resuscitation Council UK’s Recommended Summary Plan for Emergency Care and Treatment (ReSPECT) forms.

Methods

We sampled the hip fracture population at Burton Hospital orthopaedic ward on random days between October and November 2024. We separately collected data on other patients with a ReSPECT form to see whether omissions on these forms were specific to the hip fracture group or replicated more widely.

Results

We collected data for 42 patients, including 33 patients admitted with hip fractures and nine non-hip fracture patients. We found that 20 (61%) hip fracture patients had a ReSPECT form that documented the appropriateness of CPR. However, only nine (45%) of these documented an appropriate CoT. This left only nine (27%) hip fracture patients with a full TEP. Out of all decisions documented, 16 (80%) were endorsed by a consultant.

In the non-hip fracture group, we found that many ReSPECT forms also lacked a CoT. There was no significant difference in the completion of a CoT on ReSPECT forms between the hip fracture and non-hip fracture groups (p=0.69).

Conclusion

We found that many patients with a hip fracture did not have a documented recommendation on the appropriateness of CPR, and even fewer had a decision documented on the appropriateness of escalation. This meant that neither area met the standard of 100% set for our audit. This was in part due to the incompletion of the clinical recommendations section on the ReSPECT forms, which we highlight is an often-omitted section in our hospital. Review of the escalation plan within a comprehensive geriatric assessment, and increasing clinician education on TEPs, may help to improve the number of complete treatment escalation plans made.

## Introduction

Each year in the UK, there are an estimated 70,000 hip fractures [1]. It is a fracture that disproportionately affects elderly and frail patients, with the majority being over 80 years old and often with many pre-existing co-morbidities [1,2]. Given this, it is unsurprising that this patient group often have complex medical, surgical and rehabilitation requirements, requiring a multidisciplinary approach to ensure the best possible outcome [3]. Fragility fractures as a whole cost the UK a total of £4.4 billion, with hip fractures making approximately 45% of this figure [4].

Mortality following a hip fracture is very high, standing overall at 10% at one month and 30% at one year [2]. For those who develop post-operative complications, the prognosis is considerably worse. Post-operative chest infections have a one-month mortality of 43%, and heart failure carries an extremely high mortality with 92% dying within one year [2].

The British Orthopaedic Association (BOA) recommends that ceilings of treatment (CoT) should be discussed pre-operatively at the consultant level for all elderly and frail trauma patients [5]. This includes the appropriateness of escalation and cardiopulmonary resuscitation (CPR). Treatment escalation plans (TEPs) are a guide as to which life-sustaining interventions (e.g. invasive ventilation) should or should not be explored in the event of deterioration and can help prevent inappropriate interventions [6]. While decisions on CPR form part of this decision, TEPs also encompass the appropriateness of other life-prolonging interventions, and escalation to areas such as a high dependency unit (HDU) or intensive care unit (ICU) [6,7]. When decisions regarding CoT are not documented, this can lead to difficult decision-making should the patient deteriorate. Many of these deteriorations occur in out-of-hours settings, and TEP completion has been shown to reduce the amount of inappropriate out-of-hours care [8]. TEP completion has also been linked to reducing inappropriate escalation to ICU, thereby reducing the stress and discomfort of inappropriately pursuing invasive treatment and investigation [9]. TEPs are generally viewed as a positive part of hospital care by patients when completed, alongside providing clear communication to the out-of-hours teams as to what interventions would or would not be appropriate to explore [7].

The Recommended Summary Plan for Emergency Care and Treatment (ReSPECT) form is a way of documenting discussions with a patient on their goals of care and the agreed focus of their care [10]. It contains a section to outline recommendations for realistic CoTs and what specific treatments would or would not be appropriate. There is also a separate section specifically to document whether or not CPR is recommended. The form is designed to follow on from a ReSPECT discussion where these topics are sensitively addressed and explained [10]. Importantly, the form is not a legally binding document but rather a summary of recommendations in the event of a deterioration, still requiring clinical judgement depending on the specific situation [10]. It is the standard practice in our hospital that discussions and decisions on TEPs are recorded on ReSPECT forms.

Given the standard set by the BOA, we devised an audit to see how many hip fracture patients had a completed TEP (including recommendations on CPR and CoT) in the form of a ReSPECT form during their inpatient stay. We also wanted to assess how many recommendations on CPR were being made without a wider recommendation on CoT being documented at the same time. We therefore also looked at data on other orthopaedic patients who did not have a hip fracture but did have a ReSPECT form to assess how many of these forms had a completed CPR recommendation but lacked recommendations on their wider CoT.

## Materials and methods

Study design

The inpatient hip fracture population on the orthopaedic ward at Burton Hospital was anonymously recorded on several randomly selected days from October to November 2024. The following data were recorded: age, whether a ReSPECT form had been completed, the CPR status recommended on the form, whether any further clinical recommendations on appropriateness of escalation were included, and what CoT was specified. The grade of the most senior clinician endorsing the form was also recorded.

Separately, the same data for orthopaedic patients with a ReSPECT form and admission for a reason other than a hip fracture was recorded on the same days. Their reason for admission was also recorded. This was to assess whether any trends or omissions in the forms were specific to patients with hip fractures or seen more widely in ReSPECT form completion.

Standards

The outcomes were analysed against the following standards derived from the BOA (Table 1).

**Table 1:** Audit standards Table independently developed by the authors based on publicly available audit standards derived from the British Orthopaedic Association guidance on the care of elderly or frail trauma patients [5]. CPR = cardiopulmonary resucitation

Outcome	Standard
Hip fracture patients with a documented recommendation on the appropriateness of CPR	100%
Hip fracture patients with a documented recommended ceiling of treatment	100%
Above decisions being endorsed by a consultant	100%

Statistical analysis

Data were analysed using Microsoft Excel (Microsoft Corp., Redmond, WA, USA). Statistical testing between the two groups was carried out using either the Chi-squared test or Fisher’s exact test, where appropriate. Where Fisher’s exact test was used, the odds ratio provided is the conditional maximum likelihood estimate of the odds ratio.

Ethics

This study was performed as a local audit within University Hospitals Derby and Burton NHS Foundation Trust, UK. It did not meet the criteria for research; therefore, it did not require additional ethical approval. It was a locally registered audit (number UHDBS476).

## Results

Overall, we collected data for 42 patients, including 33 patients admitted with hip fractures and nine patients with an alternative diagnosis.

Hip fracture group

The average age of the hip fracture group at the time of admission was 85.5 years (range = 71-92 years), which is in keeping with previous studies on this population [1].

**Figure 1:**
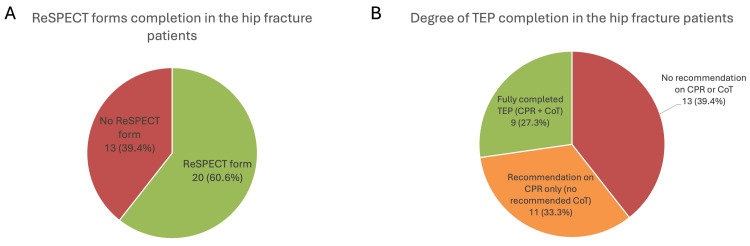
Summary of treatment escalation plan completion for the 33 patients with a hip fracture (A) A pie chart showing the proportions of patients with a hip fracture with and without a completed ReSPECT form. (B) A pie chart showing the proportions of hip fracture patients with complete, incomplete and no TEP documented. CoT = ceiling of treatment, CPR = cardiopulmonary resuscitation, ReSPECT = Recommended Summary Plan for Emergency Care and Treatment, TEP = treatment escalation plan

In total, 20 (60.6%) patients with a hip fracture had a ReSPECT form completed in their clinical notes, and 13 (39.4%) had no ReSPECT form (Figure 1A). Of the 20 completed ReSPECT forms, nine (45.0%) had a documented CoT within the clinical recommendations section, and 11 (55.0%) had no documented CoT, with this section left completely blank.

This left nine hip fracture patients (27.3%) with a fully documented TEP and 24 (72.7%) with either an incomplete TEP (in the form of a CPR recommendation but no recommendation on CoT) or no TEP at all (no ReSPECT form completed; Figure 1B).

**Figure 2:**
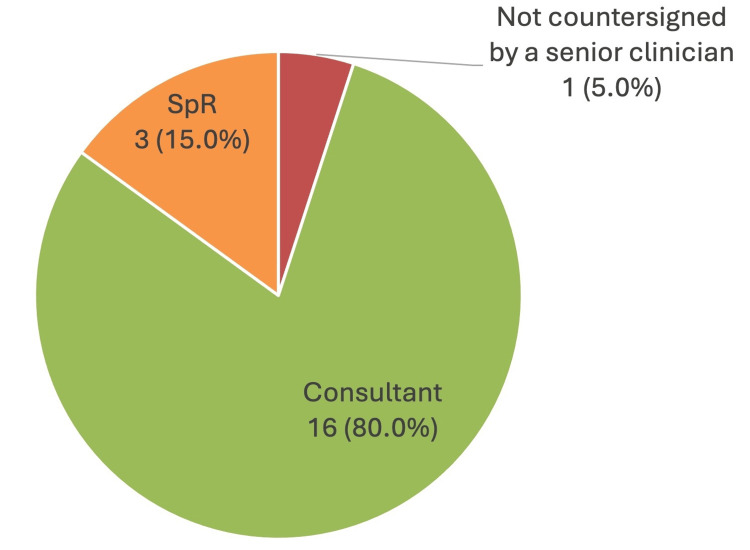
Pie chart showing the most senior endorsing clinician on the 20 ReSPECT forms for hip fracture patients ReSPECT = Recommended Summary Plan for Emergency Care and Treatment, SpR = speciality registrar

In terms of clinician endorsement of the ReSPECT form recommendations, 16 (80.0%) of the forms were endorsed by a consultant, three (15.0%) were endorsed by a speciality registrar, and one (5.0%) had not been endorsed by any senior clinician (Figure 2).

**Figure 3:**
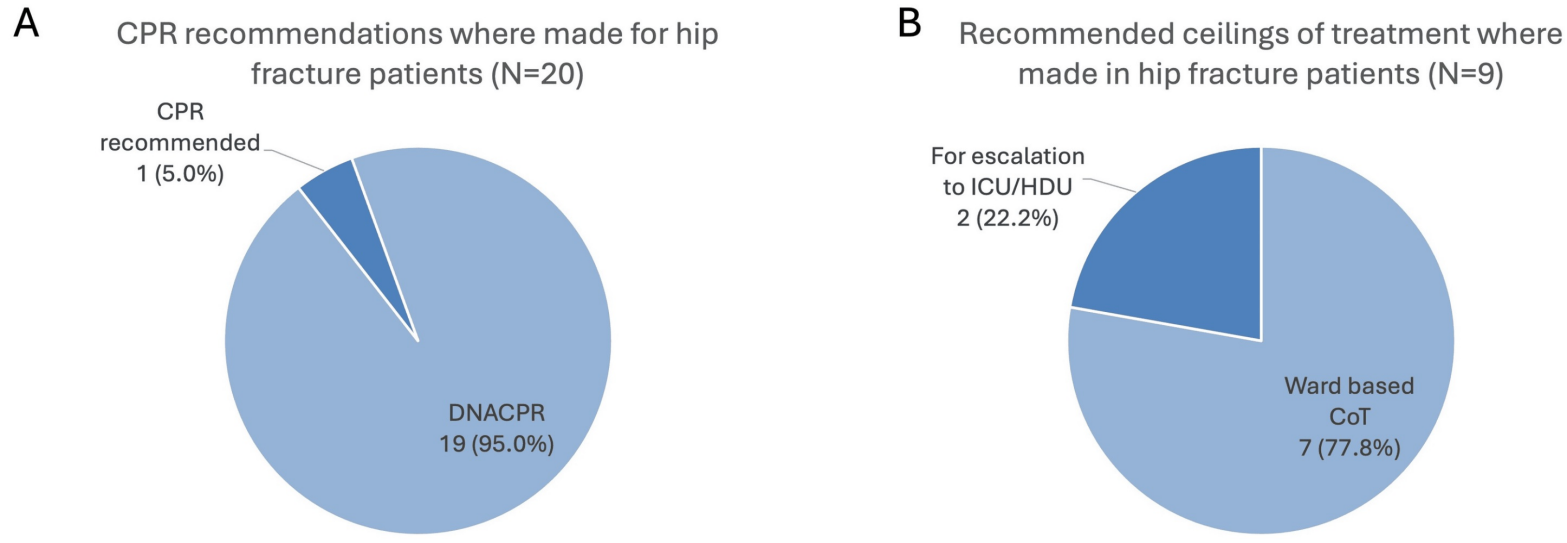
Summary of the clinical recommendations made for hip fracture patients (A) Pie chart showing the CPR recommendation provided on the ReSPECT form in the 20 hip fracture patients where a recommendation on CPR was given. (B) Pie chart showing the recommended ceiling of treatment provided on the ReSPECT form in the nine hip fracture patients where a recommendation was given. CoT = ceiling of treatment, CPR = cardiopulmonary resuscitation, DNACPR = do not attempt resuscitation, HDU = high dependency unit, ICU = intensive care unit, ReSPECT = Recommended Summary Plan for Emergency Care and Treatment

All 20 ReSPECT forms included a recommendation on whether to attempt CPR, with 19 (95.0%) having a recommendation of “Do Not Attempt CPR” (DNACPR) and one (5.0%) recommending CPR in the event of cardiac arrest (Figure 3). Of the nine patients that included a documented CoT (thus having a fully completed TEP), seven (77.8%) recommended a ward-based CoT and two (22.2%) recommended consideration of escalation for treatments in ICU or HDU (Figure 3).

Table 2 shows our results compared against the predefined standards.

**Table 2:** Audit results compared to the pre-defined standards Audit standards were derived from the British Orthopaedic Association’s guidance on the elderly or frail trauma patient compared to the results [5]. CPR = cardiopulmonary resuscitation

Outcome	Standard	Result: N (%)
Hip fracture patients with a documented recommendation on the appropriateness of CPR	100%	20 (61%)
Hip fracture patients with a documented recommended ceiling of treatment	100%	9 (27%)
Above decisions being endorsed by a consultant	100%	16 (80%)

Non-hip fracture group

**Figure 4:**
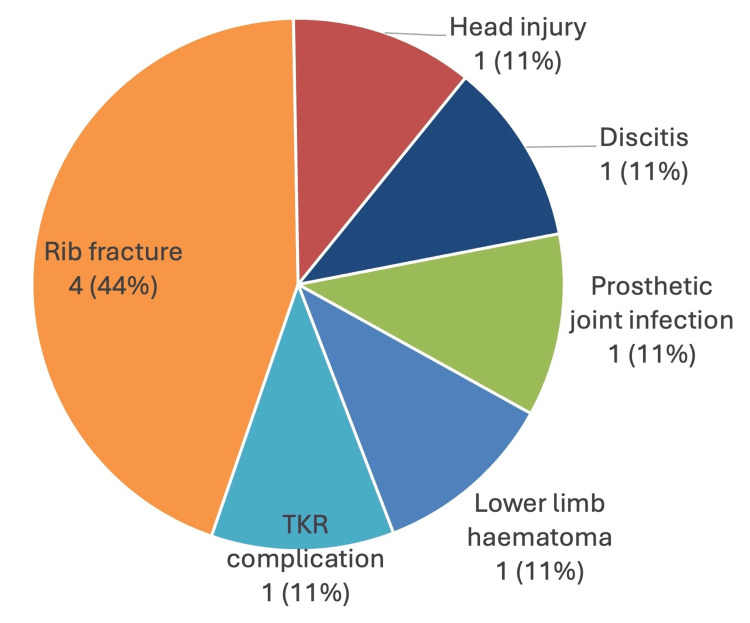
Pie chart showing the diagnoses for all nine of the non-hip fracture patients TKR = total knee replacement

There were nine non-hip fracture trauma patients, of which the majority were admitted for rib fractures, but also included patients admitted for several other diagnoses (Figure 4).

**Figure 5:**
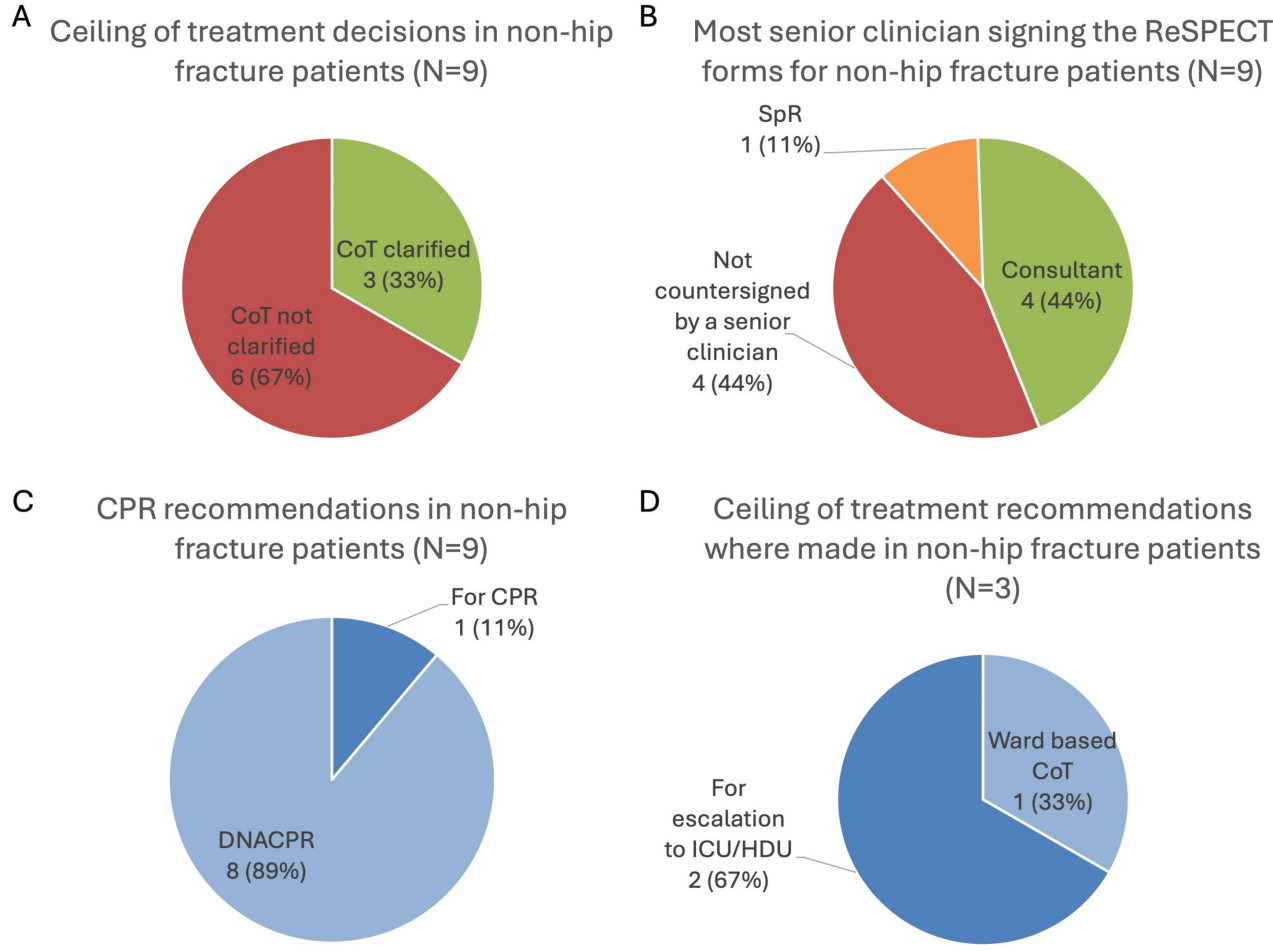
Summary of the results for the non-hip fracture patients (A) Pie chart showing the proportion of the nine non-hip fracture patients with a documented ceiling of treatment. (B)  Pie chart showing the most senior endorsing clinician of the ReSPECT form for the nine non-hip fracture patients. (C) Pie chart showing the recommendations on CPR for the non-hip fracture patients included on the nine ReSPECT forms. (D) Pie chart showing the recommended ceiling of treatment for the non-hip fracture patients on the three ReSPECT forms, where this was clarified. CoT = ceiling of treatment, CPR = cardiopulmonary resuscitation, HDU = high dependency unit, ICU = intensive care unit, ReSPECT = Recommended Summary Plan for Emergency Care and Treatment, SpR = speciality registrar

Due to the way these patients were collected, all nine had a ReSPECT form completed. The proportion of completed CoTs was similar to the hip-fracture group, with three (33.3%) providing a recommended CoT, and six (66.7%) making no recommendation (Figure 5A). There was no significant difference when comparing the hip fracture to the non-hip fracture group in the proportion of ReSPECT forms with a recommended CoT using Fisher's exact test (p=0.694; Table 3). Fisher’s exact test was used, given the small sample sizes.

**Table 3:** Statistical results comparing ReSPECT forms between the hip fracture and non-hip fracture groups 95% CI = 95% confidence interval, CoT = ceiling of treatment, ReSPECT = Recommended Summary Plan for Emergency Care and Treatment * The conditional maximum likelihood estimate of the odds ratio is given as Fisher’s exact test was used in all cases.

Category for comparison	Results for the hip fracture group (%)	Results for the non-hip fracture group (%)	Conditional maximum likelihood estimate of the odds ratio* (95% CI)	P-value for Fisher’s exact test
ReSPECT forms with a completed CoT	11/20 (45%)	6/9 (67%)	1.61 (0.25 – 12.83)	0.694
ReSPECT forms with a consultant signature	16/20 (80%)	4/9 (44%)	4.68 (0.67 – 38.29)	0.088

Senior clinician endorsement was lower than the hip-fracture group, with four (44.4%) forms endorsed by a consultant, one (11.1%) endorsed by a speciality registrar, and four (44.4%) not endorsed by a senior clinician (Figure 5B). Although there were fewer consultant endorsements compared to the hip fracture group, the difference was not significant using Fisher's exact test (p=0.088; Table 3). Fisher’s exact test was again chosen due to the small sample sizes.

In terms of the decisions on these nine ReSPECT forms, eight (88.9%) recommended DNACPR, with only one (11.1%) recommending CPR (Figure 5C). Of the three forms where a CoT was clarified, one (33.3%) recommended a ward-level CoT and two (66.7%) recommended consideration of escalation to ICU/HDU (Figure 5D).

## Discussion

In this single-centre study, none of the predefined audit standards was met. In total, 61% of patients with a hip fracture had a decision made on whether CPR would be appropriate, falling short of the expected standard. Furthermore, even for those where a CPR decision was made, 55% of these did not have a documented CoT, with the section for clinical recommendations left uncompleted. This suggests that while clinicians are more likely to discuss and decide on the appropriateness of CPR, CoT and escalation were addressed less often, leading to incomplete TEPs. Similar proportions of ReSPECT forms in the non-hip fracture patients had a blank clinical recommendations section (meaning there was no documented CoT) compared to the hip fracture patients, thus highlighting this as a generally overlooked part of the form.

The vast majority (93%) of the ReSPECT forms completed in the hip fracture patients were endorsed by a senior clinician (speciality registrar and above - i.e., Speciality Trainee 3 and above), and 80% were endorsed by a consultant. This falls just short of the high standard expected of 100%. Part of this may be due to our collection method of cross-sectional samples on randomly chosen days. It is possible that some of these forms had only recently been completed and therefore would go on to be endorsed by a consultant soon after they were sampled.

Previous studies looking at ReSPECT forms have shown varying degrees of completion of the clinical recommendations section. One trust-wide study found that 39% of ReSPECT forms left the clinical recommendations section completely blank [11], which is relatively comparable with our own incompletion rate of 55% within the hip fracture patients. Other studies on this topic have seen notably lower incompletion rates than this, below 22% [12,13].

Incomplete ReSPECT forms can be due to incomplete discussion with patients and their families. This can be due to patients wanting time to consider options, as well as time restraints upon the clinician, limiting the conversation [14]. However, incomplete discussions can still be useful as they may help catalyse future conversations; therefore, they should take less time when revisited. The timing of the TEP discussion is very important. Many of the ReSPECT discussions that happen in our department occur at admission and the initial clerking. Admission and initial clerking are often times when the patient’s family is present, and the patient may wish for them to be part of these discussions. However, it can also be a point where other clinical factors, such as establishing pain relief or explaining the diagnosis and its management, may make it necessary to delay TEP discussions. This may explain why we found many TEPs left incomplete, as time restraints and other clinical demands are often high at this point. Therefore, having other occasions in the patient journey where TEPs are reviewed and discussed, such as when they are consented for theatre, is important. However, these patients also require timely surgery and fixation of their fracture, with the target time in the UK set at within 36 hours from admission [5]. Given this, it was not mandatory to discuss the wider TEP or CoT during the informed consent process within our department at the time of data collection; however, it was encouraged.

Where a decision was documented on the CoT, the vast majority recommended a ward-level CoT, with only a minority recommending consideration of treatments in ICU or HDU. This reflects the frailty of the hip fracture population in general. As described previously, hip-fracture patients have high morbidity and mortality, with their outcomes becoming considerably worse should they develop a post-operative complication [2]. This highlights the severity of post-operative complications in this group. Importantly, many of these complications (such as chest infections and heart failure) may not be directly related to the surgery itself, yet still carry high mortality. Therefore, decisions on the CoT are very important, as it is quite possible that patients in this group can become extremely unwell even if the surgery itself is uneventful.

It is important to mention again that the treatment of frail and elderly trauma patients (of which hip fracture patients make up a large proportion) requires a multidisciplinary approach [5]. Decisions and discussions on CoT, including the appropriateness of specific treatments in the event of deterioration, can at times be difficult and complex, and the BOA recommend they should be jointly discussed by the treating teams [5]. They also recommend that all elderly or frail trauma patients should have a comprehensive geriatric assessment (CGA) within 72 hours [5]. This should include assessment of future wishes, which therefore may also include decisions on CoT [15,16]. Part of a CGA will almost always include collaboration with the orthogeriatric team, and it is therefore worth considering integration and sharing of TEP decision-making into their review. At the time of data collection, the local orthogeriatric review did not routinely include review of TEPs. If included, this would likely increase the number of TEP recommendations made, helping to increase this closer to the target standards set, and help guide decisions in the event of unexpected deterioration.

Previous studies looking at increasing TEP completion in hip fracture patients have shown improvements using educational sessions for clinicians [17,18]. This is perhaps because one barrier to initiating TEP conversations is unfamiliarity with the process, or the misconception that the discussion should be limited purely to whether CPR is recommended or not. It is also possible that clinicians may believe TEPs lead to worse outcomes for the patient by limiting the care they receive. However, having a DNACPR recommendation has been shown not to be an independent factor for mortality [19]. Furthermore, a TEP can recommend CPR and escalation for invasive treatment, should that be the outcome of the discussion. Therefore, there is no reason to believe that having a ReSPECT form should limit treatment options, and rather may help on-call teams quickly recognise when escalation should be considered. TEPs have been shown to reduce the number of non-beneficial interventions and reduce patient harm, therefore leading to better patient care [20]. They have also been found to be helpful in guiding treatment for on-call teams who may have limited knowledge of the patient beforehand [21].

Limitations

The main limitation of this audit was that we did not record whether the hip fracture patients were pre-operative or post-operative in their patient journey. This, therefore, does mean some of the hip fracture patients may have gone on to have had a ReSPECT form and subsequent TEP completed following data collection but prior to surgery (and so technically meet the set standard).

A second, smaller limitation is that we only looked at TEP documentation in the form of ReSPECT forms. It is possible that a TEP could have been documented in other places within the patient notes, and therefore possible that this audit failed to capture all TEP documentation. However, within our hospital, it is standard practice to record TEP and CPR recommendations within a ReSPECT form, and therefore, it would be rare for these decisions to be recorded elsewhere. Furthermore, decisions on CoT and CPR are only useful if they are accessible at the time they are needed. Given that the standard practice in our hospital is to record these decisions in a ReSPECT form, decisions recorded elsewhere would be less likely to be found or accessed.

## Conclusions

We found that 61% of patients admitted with a hip fracture had a documented recommendation on CPR, and 27% had a fully documented TEP (including a documented CoT), both falling below the 100% expected audit standard. This highlights that a large proportion of ReSPECT forms did not have the clinical recommendations of care section completed in our department. We found no significant difference in the likelihood of completion of the CoT section of ReSPECT forms between hip fracture and non-hip fracture groups, highlighting that this, in general, appears to be an often-omitted section of the form. We further found that 80% of ReSPECT forms completed in hip fracture patients were endorsed by a consultant, meaning that these important decisions were generally taken with the approval of senior clinical personnel, although this fell just short of the 100% audit standard.

Treatment of the frail and elderly trauma patient requires a multidisciplinary approach, including a CGA. Improvements in the number of completed TEPs may be made by integrating these discussions into part of the routine orthogeriatric review and through educational sessions aimed at clinicians of all stages.

## References

[1] Royal College of Physicians (2026). Room for improvement: hip fracture care in 2024. Room for Improvement: Hip Fracture Care in 2024.

[2] Roche JJ, Wenn RT, Sahota O, Moran CG (2005). Effect of comorbidities and postoperative complications on mortality after hip fracture in elderly people: prospective observational cohort study. BMJ.

[3] Office for Health Improvement and Disparities (2026). Falls: Applying All Our Health. Falls: Applying All Our Health..

[4] Riemen AH, Hutchison JD (2016). The multidisciplinary management of hip fractures in older patients. Orthop Trauma.

[5] (2026). BOA standard: the care of the older or frail orthopaedic trauma patient. https://www.boa.ac.uk/static/a30f1f4c-210e-4ee2-98fd14a8a04093fe/boast-frail-and-older-care-final.pdf.

[6] Sayma M, Nowell G, O'Connor A (2018). Improving the use of treatment escalation plans: a quality-improvement study. Postgrad Med J.

[7] Obolensky L, Clark T, Matthew G, Mercer M (2010). A patient and relative centred evaluation of treatment escalation plans: a replacement for the do-not-resuscitate process. J Med Ethics.

[8] Stockdale C, Trivedi B, Jerome E (2014). Implementation of a combined cardiopulmonary resuscitation and treatment escalation plan document in a district general hospital. BMJ Qual Improv Rep.

[9] Fadel MG, Parekh K, Hayden P, Krishnan P (2018). Improving resuscitation decisions: a trust-wide initiative. BMJ Open Qual.

[10] Fritz Z, Slowther AM, Perkins GD (2017). Resuscitation policy should focus on the patient, not the decision. BMJ.

[11] Anik E, Hurlow A, Azizoddin D (2024). Characterising trends in the initiation, timing, and completion of recommended summary plan for emergency care and treatment (ReSPECT) plans: Retrospective analysis of routine data from a large UK hospital trust. Resuscitation.

[12] Hawkes CA, Griffin J, Eli K (2022). Implementation of ReSPECT in acute hospitals: a retrospective observational study. Resuscitation.

[13] Huxley CJ, Eli K, Hawkes CA (2024). Are completed ReSPECT plans facilitating person-centred care? An evaluation of completed plans in UK general practice. Resusc Plus.

[14] Eli K, Huxley CJ, Hawkes CA, Perkins GD, Slowther AM, Griffiths F (2022). Why are some ReSPECT conversations left incomplete? A qualitative case study analysis. Resusc Plus.

[15] Turner G, Clegg A (2014). Best practice guidelines for the management of frailty: a British Geriatrics Society, Age UK and Royal College of General Practitioners report. Age Ageing.

[16] (2026). Comprehensive Geriatric Assessment (CGA): future wishes. https://www.bgs.org.uk/cga-future-wishes.

[17] Heylen J, Kemp O, Macdonald NJ, Mohamedfaris K, Scarborough A, Vats A (2022). Pre-operative resuscitation discussion with patients undergoing fractured neck of femur repair: a service evaluation and discussion of current standards. Arch Orthop Trauma Surg.

[18] Misselbrook GP, Jackman D, Vora C, Briant-Evans T, Wilkinson A (2020). Implementing and improving the ReSPECT process within medical and orthopaedic departments of a district general hospital. Prog Palliat Care.

[19] Simons AE, Karres J, Nijland LM, Ultee JM, Kerkhoffs GM, Vrouenraets BC (2017). Do-not-resuscitate orders and early mortality in hip fracture patients. Age Ageing.

[20] Lightbody CJ, Campbell JN, Herbison GP, Osborne HK, Radley A, Taylor DR (2018). Impact of a treatment escalation/limitation plan on non-beneficial interventions and harms in patients during their last admission before in-hospital death, using the Structured Judgment Review Method. BMJ Open.

[21] Shermon E, Munglani L, Oram S, William L, Abel J (2017). Reducing DNACPR complaints to zero: designing and implementing a treatment escalation plan using quality improvement methodology. BMJ Open Qual.

